# Using Routine Surveillance Data to Estimate the Epidemic Potential of Emerging Zoonoses: Application to the Emergence of US Swine Origin Influenza A H3N2v Virus

**DOI:** 10.1371/journal.pmed.1001399

**Published:** 2013-03-05

**Authors:** Simon Cauchemez, Scott Epperson, Matthew Biggerstaff, David Swerdlow, Lyn Finelli, Neil M. Ferguson

**Affiliations:** 1 MRC Centre for Outbreak Analysis and Modelling, Department of Infectious Disease Epidemiology, Imperial College London, London, United Kingdom; 2 Centers for Disease Control and Prevention, Atlanta, Georgia, United States of America; The University of Hong Kong, Hong Kong

## Abstract

**Background:**

Prior to emergence in human populations, zoonoses such as SARS cause occasional infections in human populations exposed to reservoir species. The risk of widespread epidemics in humans can be assessed by monitoring the reproduction number *R* (average number of persons infected by a human case). However, until now, estimating *R* required detailed outbreak investigations of human clusters, for which resources and expertise are not always available. Additionally, existing methods do not correct for important selection and under-ascertainment biases. Here, we present simple estimation methods that overcome many of these limitations.

**Methods and Findings:**

Our approach is based on a parsimonious mathematical model of disease transmission and only requires data collected through routine surveillance and standard case investigations. We apply it to assess the transmissibility of swine-origin influenza A H3N2v-M virus in the US, Nipah virus in Malaysia and Bangladesh, and also present a non-zoonotic example (cholera in the Dominican Republic). Estimation is based on two simple summary statistics, the proportion infected by the natural reservoir among detected cases (*G*) and among the subset of the first detected cases in each cluster (*F*). If detection of a case does not affect detection of other cases from the same cluster, we find that *R* can be estimated by 1−*G*; otherwise *R* can be estimated by 1−*F* when the case detection rate is low. In more general cases, bounds on *R* can still be derived.

**Conclusions:**

We have developed a simple approach with limited data requirements that enables robust assessment of the risks posed by emerging zoonoses. We illustrate this by deriving transmissibility estimates for the H3N2v-M virus, an important step in evaluating the possible pandemic threat posed by this virus.

*Please see later in the article for the Editors' Summary*

## Abstract 2

Using a novel method to assess the risks of outbreaks and epidemics, Simon Cauchemez and colleagues provide insight into a simple tool that allows for more robust monitoring of the epidemic potential of zoonoses.

## Editors' Summary

**Background:**

When a virus emerges in the human population, such viruses can cause global epidemics potentially harming large numbers of people. Zoonotic viruses are viruses that are transmissible from animals to humans; the global health threat of zoonotic viruses was recently demonstrated by the 2009 H1N1 influenza pandemic and the SARS epidemic in 2003. Many zoonotic viruses are transmitted by means of an infected vector, while others can be transmitted by inhalation, contact with infected excretions, or by direct contact with an infected animal. Zoonotic viruses primarily cause occasional infections in human populations exposed to reservoir species (the animal species harboring the virus) because the pathogens are usually poorly adapted for sustained human-to-human transmission. However, zoonotic viruses are under strong selective pressure to acquire the ability for human-to-human transmission.

**Why Was This Study Done?:**

The highly pathogenic H5N1 avian influenza epidemic was alarming to many because of the high mortality rate in humans and its rapid spread in avian populations. Public health response to outbreaks such as those of H5N1 avian influenza and SARS required reliable estimates of transmissibility (how easily it spreads between people) and severity (the proportion of infected people who needed hospital treatment). For efficient prevention and control of the emerging epidemic, quantitative and rigorous assessment of the associated risks is needed. Specifically, health officials and researchers need fast, reliable methods for estimating the extent to which a virus has acquired the ability to transmit from person to person. In this study, the authors developed a novel method to estimate a standard measure of transmissibility, the human-to-human reproduction number *R* (average number of persons infected by a human case) of a zoonotic virus, which overcomes many of the limitations of existing methods.

**What Did the Researchers Do and Find?:**

The authors developed a simple method to estimate the reproduction number of emerging zoonoses from routine surveillance data. By using two simple summary statistics, the proportion infected by the natural reservoir among detected cases (*G*) and among the subset of the first detected cases in each cluster (*F*), the authors estimated R, the reproduction number of zoonoses in humans. The authors then applied their new approach to assess the human-to-human transmissibility of swine-origin influenza A variant (H1N1v, H1N2v, and H3N2v) virus, in particular that of the H3N2v-M virus, from US surveillance data for the period December 2005–December 2011, Nipah virus in Malaysia and Bangladesh, as well as to a non-zoonotic pathogen Vibrio Cholerae in the Dominican Republic. This study demonstrates the applicability of this novel approach to estimating R during zoonotic and certain non-zoonotic outbreaks.

**What Do These Findings Mean?:**

Cauchemez and colleagues show that their new approach will be useful in assessing human-to-human transmissions during zoonotic outbreaks. The authors show that their new method does not require as much of an investigation effort as existing methods, the statistical treatment of the data is extremely simple, and the robustness of the method is demonstrated even if larger clusters are more likely to be detected and if the ability to detect all cases in a cluster once a cluster is identified is low. This method of estimating R is designed for the context of subcritical outbreaks, i.e., *R*<1. However if *R*≥1, other estimation methods will be needed.

**Additional Information:**

Please access these Web sites via the online version of this summary at http://dx.doi.org/ 10.1371/journal.pmed.1001399.

- The US Centers for Disease Control and Prevention's (CDC) National Center for Emerging and Zoonotic Infectious Diseases provides information on infectious disease
- The CDC also has resources for pandemic flu and H3N2v
- The University of Wisconsin's School of Veterinary medicine has an online tutorial on zoonotic diseases
- The European Food Safety Authority (EFSA) also provides comprehensive information on zoonotic diseases

## Introduction

The 2009 A(H1N1)pdm09 influenza pandemic [1], the SARS epidemic in 2003 [2], and the recent emergence of a novel coronavirus [3] are recent reminders of the global health threat posed by zoonotic viruses. Prior to widespread emergence in human populations, such pathogens can cause occasional infections in sub-populations that have been exposed to reservoir species (common reservoir species include for example bats, birds, swine, non-human primates). Whilst viruses causing such “spill-over” infections are usually poorly adapted for sustained human-to-human transmission, they are under strong selection pressure to increase transmissibility once in humans [4]. If the reproduction number *R* (i.e., the average number of persons infected by a case) evolves to exceed 1, a large scale epidemic in humans may result. Over the last decade, particular concerns were raised regarding highly pathogenic H5N1 avian influenza, due to the high mortality rate seen in humans and the virus's rapid spread in avian populations. However, as the A(H1N1)pdm09 influenza pandemic demonstrated, H5N1 is not the only influenza virus that may pose a pandemic risk. Recently, a swine-origin triple reassortant influenza A(H3N2) variant virus has emerged in the United States, carrying the matrix gene (M) from the H1N1pdm09 virus (H3N2v-M) [2]–[4]. Studies in animal models have suggested that the presence of the H1N1pdm09 M gene may increase transmissibility of the virus [5],[6]. From January 2012 to September 2012, 307 laboratory-confirmed H3N2v-M human infections were reported to Centers for Disease Control and Prevention (CDC) [5] as opposed to 12 throughout 2011 [6]. The majority of cases have been associated with agricultural fairs but there are documented events of human-to-human transmission [7]. The surge in cases observed in summer 2012 raised public health concerns [8]. Threats from zoonoses are not limited to influenza: more than half of all recent emerging infectious disease events were zoonotic [9].

**Figure 1:**
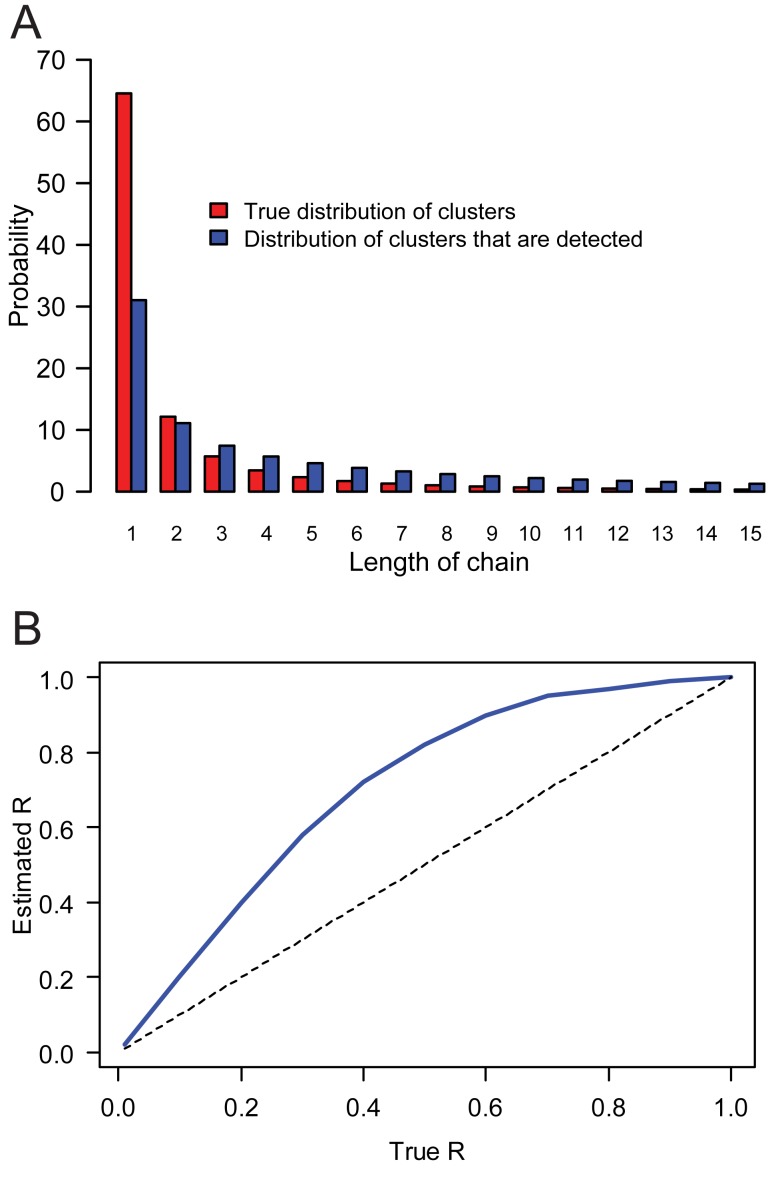
Selection bias and the estimation of the reproduction number *R*. (A) True distribution of cluster sizes (red) and distribution for clusters that are detected (blue), for *R* = 0.7. (B) Asymptotic estimate of the reproduction number as a function of the true reproduction number, derived from distribution of cluster sizes of detected clusters. The detection rate is set to ρ = 1% and the overdispersion parameter of the offspring distribution *k* = 0.5.

For efficient prevention and control, quantitative and rigorous assessment of the risks associated with emerging zoonoses is desirable—in particular the risk that an emerging pathogen evolves to cause sustained human-to-human transmission. One approach to such risk assessment is by monitoring the reproduction number *R* of zoonoses in humans, with an alarm being raised if *R* increases or approaches 1 [9]–[11]. However, until now, estimating *R* required detailed outbreak investigations of human clusters [10],[11] and suffered from three important limitations: (1) the resources, access, and expertise needed to conduct investigations is not always available; (2) the proportion of cases that are missed during outbreak investigations may vary by setting and be difficult to assess; (3) even if the study is complete, the data collection process can be affected by a selection bias whereby larger outbreaks are more likely to be detected so that estimates of transmissibility may be biased upward. Consider for example a scenario where *R* = 0.7, where each case has the same detection probability ρ = 1%, and assume that once a cluster is detected, detailed outbreak investigation ensures that all cases in the cluster are detected. With an average size of 18.3 and a 21% probability of 1-case cluster, clusters that are detected are substantially larger than normal ones (average size: 3.3; 65% probability of 1-case cluster) (Figure 1A). As expected, this selection bias leads to *R* being overestimated as illustrated for methods that use the distribution of detected cluster sizes (Figure 1B) [10].

Here, we present a new approach to estimate *R* during spillover events, aiming to address many of the limitations of existing methods. We apply our approach to assess the human-to-human transmissibility of swine-origin influenza A variant (H1N1v, H1N2v, and H3N2v) virus, in particular that of the H3N2v-M virus, from US surveillance data for the period December 2005–December 2011. We also present applications to another zoonotic virus (Nipah virus in Malaysia and Bangladesh) as well as to a non-zoonotic pathogen (Vibrio Cholerae in the Dominican Republic).

## Materials and Methods

### Ethics Statement

This investigation was determined to be part of public health response and was not considered to be human research in accordance with federal human individual protection regulations. Thus, approval from an institutional review board was not required.

### Definitions

We define a “chain of transmission” as a single reservoir-to-human transmission event followed by subsequent human-to-human transmission events (if any). A “cluster” of related cases is defined as an outbreak that takes place in a specific location and at a specific time (e.g., outbreak in fair X in August 2011), and can be composed of several chains of transmission (i.e., when a number of people are exposed to the same zoonotic source of infection).

Our method relies on the observation that the proportion of detected cases that are infected by the reservoir is largely determined by the reproduction number for human-to-human transmission, *R*. For example, for the H1N1pdm09 virus which had *R*>1 and was therefore generating long and expanding chains of transmission, none of the detected human cases were linked to the original swine reservoir. In contrast, for H5N1 highly pathogenic avian influenza, where *R* in human populations is considerably below 1, a substantial proportion of cases can be linked to exposure to the animal reservoir.

### Surveillance Scenarios

We consider two surveillance scenarios: (1) “Routine sentinel surveillance alone” where we assume that each case has a probability ρ of being detected by routine sentinel surveillance; (2) “Routine sentinel surveillance triggering outbreak investigation”, where we again assume that each case initially has a probability ρ of being detected by routine sentinel surveillance, but that when a case is detected, this may trigger an outbreak investigation. In the latter scenario, once a case is detected in a cluster, the detection probability for other cases in the same cluster may increase.

In both scenarios we assume that there is an investigation of the case detected through routine sentinel surveillance during which the probable source of infection (natural reservoir or human-to-human) is determined. However, only in scenario 2 is there also a full-blown outbreak investigation where other cases are actively searched for. We therefore expect that it is possible to group cases into clusters in scenario 2; but such information is less likely to be available in scenario 1. The US H3N2v-M situation we examine resembles scenario 2, but we also present applications for scenario 1.

We propose to estimate *R* from two simple summary statistics, the proportion infected by the natural reservoir among detected cases (*G*) and among the subset of the first detected cases in each cluster (*F*). We now determine the relationship between *G*, *F*, and *R* and develop a statistical framework to estimate *R* from *G* (scenario 1) and from *F* (scenario 2).

### Modelling the Offspring Distribution

We denote the length of a chain of transmission by *L*. Following Lloyd-Smith et al. [12], the offspring distribution (i.e., number of persons infected by a case) is modelled with a negative binomial distribution with mean *R* and overdispersion parameter *k* (parameter *k* characterizes case-to-case variation in infectiousness). The probability that a chain is of length *L* is given by [13]:
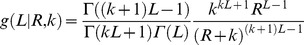
(1)


### Relationship between *G* and *R* (Scenario 1)

We first consider scenario 1 where each case has the same probability of being detected. In a typical chain of transmission of average length 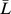 there is, by definition, one reservoir-to-human transmission event and 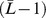 human-to-human infections. The probability *G* that a case randomly picked up by surveillance was infected by the reservoir is therefore 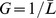. However, for subcritical outbreaks (0<*R*<1), branching process theory tells us that the average length of a chain is 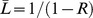 [14]. We therefore obtain 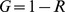.

### Relationship between *F* and *R* (Scenario 2)

We now consider surveillance scenario 2 (i.e., detection of a case may trigger an outbreak investigation). We only use data from the first detected case of each cluster to control for the change in surveillance intensity due to the outbreak investigation. We first make the stricter assumption that each cluster is made of one chain of transmission, but this assumption is relaxed in Figure S1 and Text S1. Conditional on detection of the chain, the probability *F* that the first detected case was the first case of the chain is:
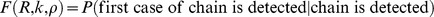


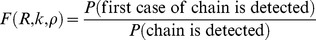


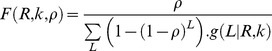
(2)When the probability of detection is small (ρ≪1), we are left again with a simple linear relationship 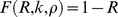 (see Text S1).

### Inference

For scenario 2, we denote the number of clusters that are investigated by *M* and the number of first detected cases of each cluster that were infected by the reservoir by *m*.

The likelihood is:
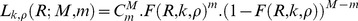
(3)Estimation is performed conditional on overdispersion parameter, *k*, and the case detection rate, ρ. We also derive bounds on *R* when *k* and ρ are unknown (see Text S1).

To account for small sample sizes, we compute the bootstrap mean estimate and the bootstrap 95% confidence intervals. We compare these estimates with those obtained under asymptotic conditions (i.e., maximum likelihood and likelihood-ratio confidence intervals) and find that they are similar (Figures S2, S3, S4; Tables S1, S2, S3; Text S1).

For scenario 1, 1−*G* is an unbiased point estimate of *R*. A simple binomial likelihood function with probability 1−*R* can be used to derive confidence intervals for *R*. These intervals capture uncertainty arising from sampling; but may underestimate other sources of uncertainty if the total number of chains of transmission (both detected and undetected) in the study population remains small (see Text S1).

### Data

Novel influenza A virus infections in humans, such as those caused by the H3N2v-M virus, are nationally notifiable conditions in the United States. When US laboratories or health care providers identify a potential infection with a novel influenza A virus they are asked to notify CDC immediately. CDC then collaborates with federal, state, and local human and animal public health colleagues to provide laboratory confirmation of the novel virus and to conduct a case and/or outbreak investigation. Outbreak investigations typically involve case investigation, contact investigations, attempts to determine potential animal or human sources of the infection, and environmental investigations. For each suspected or confirmed case detailed epidemiologic and clinical data are gathered. Notification of these cases is published in the MMWR, and in the weekly FluView national surveillance report.

From August 2011 to December 2011, CDC confirmed 12 human H3N2v-M infections [6],[7],[15],[16]. Eleven of the 12 reported cases occurred in children, most <10 years old, and three of the 12 cases were hospitalized for influenza. These 12 cases were detected in six clusters, with a median number of two cases detected per cluster (range: 1–3 cases). We assumed swine were the source of infection if there were 4 or fewer days from swine contact to onset, and that cases occurring more than 4 days after exposure to swine were likely to have been caused by human-to-human transmission. The potential human-to-human transmission events thus identified were investigated more closely to determine the likely source of infection.

## Results

### Reproduction Number *R*, Probabilities *G* and *F*

If the detection of a case does not affect detection of other cases from the same cluster (surveillance scenario 1), we find that *R* can be simply estimated as *R* = 1−*G*, where *G* is the proportion of detected cases that are infected by the reservoir. This is a general result that is independent of the case detection rate ρ and the overdispersion parameter *k* and that does not require data on clusters. It is valid as long as outbreaks are subcritical (0<*R*<1).

**Figure 2:**
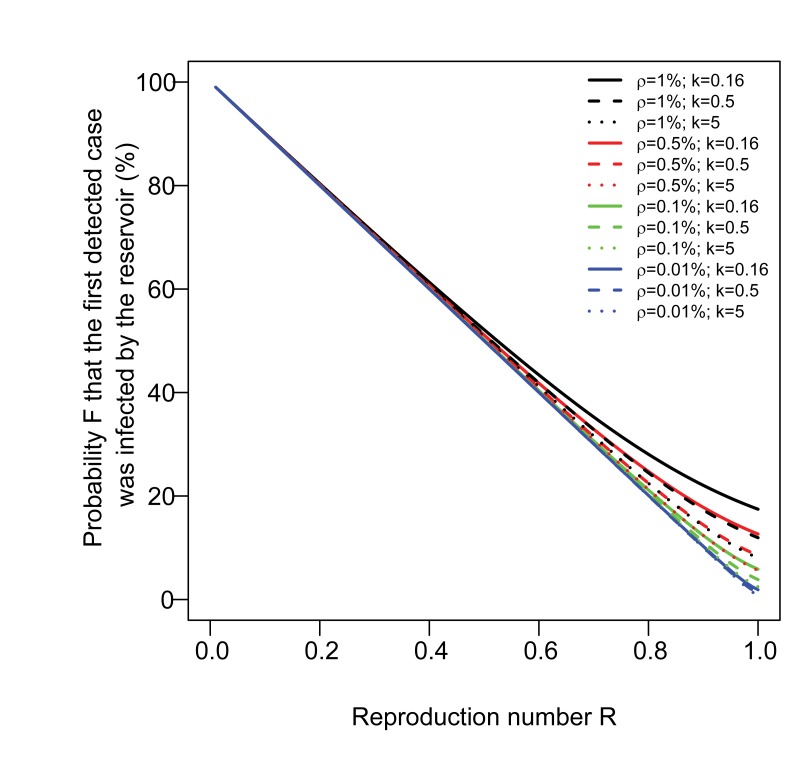
Probability *F* that the first detected case in a cluster was infected by the reservoir, as a function of the reproduction number *R*. The number of secondary cases caused by individual human cases is modelled with a Negative binomial distribution with parameters *R* and *k*, where *k* is the overdispersion parameter [12]. We consider three scenarios for the case-to-case variation in infectiousness: high (i.e., most transmission events are caused by a small proportion of cases like for SARS; *k* = 0.16), medium (*k* = 0.5), and low (*k* = 5) [12].

For situations where detection of a case may increase the probability of detecting other cases in the same cluster (surveillance scenario 2), we estimate *R* from the proportion *F* of first detected cases in each cluster that were infected by the reservoir. Figure 2 shows the relationship between the reproduction number *R* and the proportion *F* for different values of the detection rate ρ and the overdispersion parameter *k* (parameter *k* characterizes case-to-case variation in infectiousness; see [12] and Methods). When the case detection rate ρ is relatively low, at levels similar to those seen in sentinel systems like the US influenza virological surveillance network, the straight line dependence shown in Figure 2 illustrates that *R* can be estimated as *R* = 1−*F* (see Methods and Text S1). We find that it takes relatively high levels of case detection (ρ) or case-to-case variation in infectiousness (*k*) to cause substantial deviations from this linear relationship, and even then, such deviation only occurs for values of *R* close to 1 (Figure 2). We also find that the relationship is only weakly sensitive to having multiple chains of transmission per cluster of human cases (Figure S1; Text S1).

### Estimating *R* for Swine Influenza Variants

We apply our method to assess the human-to-human transmissibility of swine-origin influenza A variant (H1N1v, H1N2v, and H3N2v) virus, in particular that of the H3N2v-M virus with data collected up to December 2011. From December 2005 until December 2011, a total of 27 human clusters of swine-origin influenza A variant virus infections were investigated by the CDC in the US. In clusters caused by the H3N2v-M virus, three of six (50%) of the first detected cases were infected by swine compared with 17 of 21 (81%) in clusters caused by the other variant viruses (Table 1). Table 1 gives *R* estimates for different assumptions about the case detection rate ρ and overdispersion parameter *k*.

**Table 1:** Estimate (95% CI) of *R* for all strains, the H3N2v-M variant, and variants other than H3N2v-M, for different scenarios of detection and overdispersion in the offspring distribution.

Case Detection Rate (ρ)	Overdispersion in Offspring Distribution (*k*)	All Strains (*n* = 27, *F* = 74%)^a^	H3N2v-M Variant (*n* = 6, *F* = 50%)^a^	Variants Other Than H3N2v-M (*n* = 21, *F* = 81%)^a^
	0.16	0.35 [0.13–0.70]	0.74 [0.20>1]	0.24 [0.05–0.55]
10%	0.5	0.31 [0.12–0.57]	0.66 [0.19>1]	0.22 [0.05–0.47]
	5	0.30 [0.12–0.52]	0.60 [0.19>1]	0.22 [0.05–0.44]
	0.16	0.30 [0.12–0.55]	0.65 [0.18>1]	0.21 [0.05–0.45]
5%	0.5	0.28 [0.12–0.50]	0.58 [0.18>1]	0.21 [0.05–0.42]
	5	0.28 [0.12–0.48]	0.56 [0.18>1]	0.20 [0.05–0.41]
	0.16	0.27 [0.11–0.46]	0.54 [0.17>1]	0.19 [0.05–0.39]
1%	0.5	0.26 [0.11–0.45]	0.52 [0.17–0.91]	0.19 [0.05–0.39]
	5	0.26 [0.11–0.45]	0.51 [0.17–0.87]	0.19 [0.05–0.39]
	0.16	0.26 [0.11–0.45]	0.52 [0.17–0.92]	0.19 [0.05–0.39]
0.5%	0.5	0.26 [0.11–0.45]	0.51 [0.17–0.87]	0.19 [0.05–0.38]
	5	0.26 [0.11–0.45]	0.5 [0.17–0.85]	0.19 [0.05–0.38]
	0.16	0.26 [0.11–0.45]	0.5 [0.17–0.85]	0.19 [0.05–0.38]
0.1%	0.5	0.26 [0.11–0.45]	0.5 [0.17–0.84]	0.19 [0.05–0.38]
	5	0.26 [0.11–0.45]	0.5 [0.17–0.84]	0.19 [0.05–0.38]
	0.16	0.26 [0.11–0.44]	0.5 [0.17–0.83]	0.19 [0.05–0.38]
0.01%	0.5	0.26 [0.11–0.44]	0.5 [0.17–0.83]	0.19 [0.05–0.38]
	5	0.26 [0.11–0.44]	0.5 [0.17–0.83]	0.19 [0.05–0.38]

We consider three scenarios for the case-to-case variation in infectiousness: high (i.e., most transmission events are caused by a small proportion of cases like for SARS; k  =  0.16), medium (k  =  0.5), and low (k  =  5) [12].

a *n* is the number of clusters. *F* is the proportion of first detected cases in each cluster that were infected by the reservoir. The number of first detected cases that were infected by the reservoir was 20 for all strains, three for H3N2v-M variant, and 17 for variants other than H3N2v-M.

**Figure 3:**
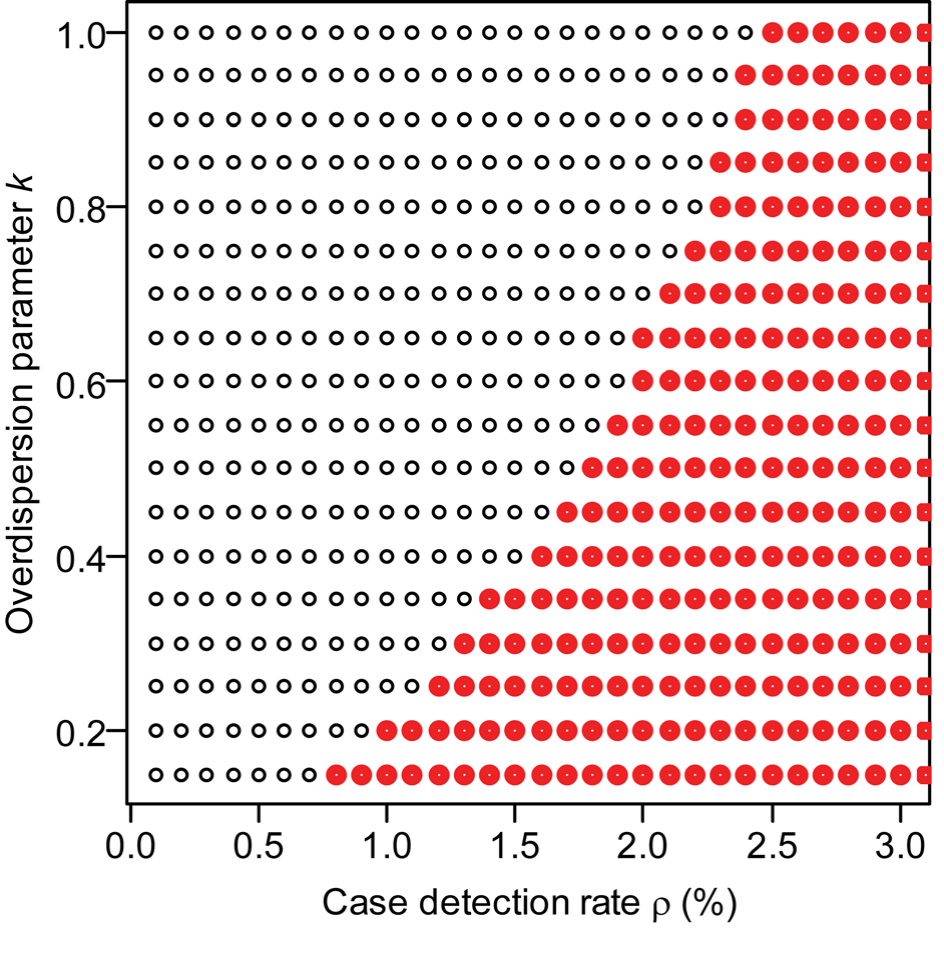
Is *R≥1* for H3N2v-M? The values of the overdispersion parameter *k* and case detection rate ρ under which we can reject the hypothesis that *R*≥1 are indicated with a black dot, and parameter values for which the assumption cannot be rejected are shown in red.

Recent efforts at CDC to evaluate the case detection rate suggest that it is low, of the order of 0.5% of all H3N2v-M-attributable cases (Biggerstaff et al., personal communication). For this likely scenario of low detection rates (≤1%), we estimate the reproduction number of variant viruses other than H3N2v-M to be 0.2 (95% CI 0.1–0.4) (Table 1). For the H3N2v-M virus, the point estimate and the lower bound of the 95% CI of *R* are 0.5 and 0.2, respectively. The upper bound of the 95% CI of *R* lies between 0.8 and >1, depending on assumptions about the case detection rate and overdispersion parameter (Table 1). If case-to-case variation in infectiousness is very large (*k* = 0.16, comparable to that seen in the SARS epidemic [12]), we can rule out the hypothesis *R*≥1 so long as the case detection rate is ≤0.7%; for a scenario with medium levels of variation (*k* = 0.5), the case detection rate must be ≤1.7% to rule out *R*≥1 (Figure 3)—a detection rate of 1.7% is unlikely to be achieved by US sentinel virologic surveillance system. The point estimate of *R* for H3N2v-M virus is more than double that of other variant viruses, but we cannot reject the hypothesis of equality (*p* = 0.15), maybe due to a lack of statistical power.

If the case detection rate is substantially higher than expected (10%), the point estimate and 95% lower bound for *R* for variant viruses other than H3N2v-M remain essentially unchanged but the upper bound increases from 0.4 to 0.4–0.6 (Table 1). The point estimate of *R* for H3N2v-M virus moves from 0.5 to 0.6–0.7.

### Is Smaller Better? A Trade-off between Bias and Precision

**Figure 4:**
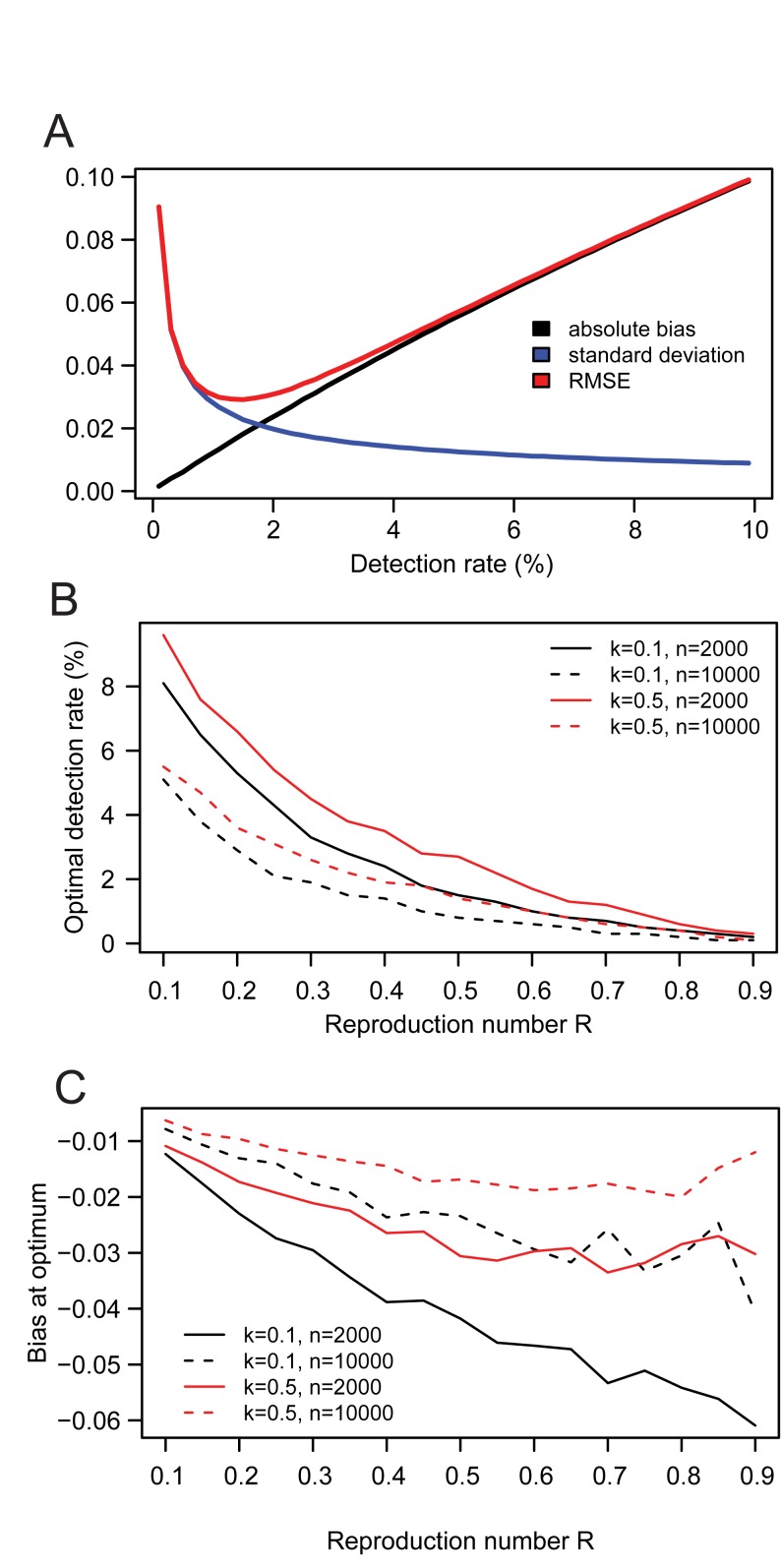
Trade-off between bias and precision for the estimator 1−*F* of the reproduction number *R*. **(A)** Absolute bias, standard deviation and root mean square error (RMSE) of estimator 1−*F* as a function of the case detection rate of the surveillance system, in a scenario with reproduction number *R* = 0.5, overdispersion parameter *k* = 0.5, and where *n* = 10,000 clusters occur in the country. Optimum trade-off between bias and precision is obtained when RMSE is minimum. (B) Optimum case detection rate as a function of the reproduction number *R*, for different values of overdispersion parameter *k* and of the number *n* of clusters. (C) Bias at the optimum case detection rate.

If the detection rate ρ is known, it is always possible to invert the relationship shown in Figure 2 to derive an unbiased estimator of *R*. However, assume that ρ is unknown and that we plan to use estimator 1−*F*, which is unbiased so long as ρ is small. As the detection rate ρ increases, so does the bias of estimator 1−*F*. However, for a fixed number of clusters occurring in the study population, larger detection rates increase precision through larger sample sizes. There is therefore a trade-off between the bias and precision of the estimator 1−*F*. This is illustrated in Figure 4A, for a scenario with reproduction number *R* = 0.5, overdispersion parameter *k* = 0.5 and where a total of 10,000 clusters occur. In this scenario, the optimum (i.e., minimizing the root mean square error) trade-off in bias versus precision is obtained for a detection rate of ρ = 1.5%. We find that the optimum detection rate for estimator 1−*F* is a decreasing function of the reproduction number *R* and the total number of clusters *n*, but an increasing function of overdispersion parameter *k* (Figure 4B). We also find that the absolute bias at the optimum detection rate remains relatively small (<0.06) (Figure 4C). In Figure S5 and Text S1, we show that thinning the data may eliminate the bias of the 1−*F* estimator, though combining information from *F* and *G* (see below) is a simpler and more convenient approach to the same goal.

### Uncertainty in the Case Detection Rate and Overdispersion Parameter

**Figure 5:**
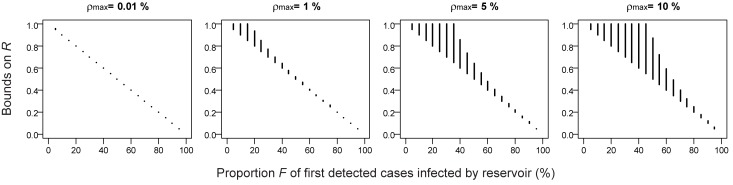
Impact of uncertainty on the case detection rate and the overdispersion parameter on estimates of the reproduction number R. 1−*F* always acts as a lower bound for *R*. Furthermore, an upper bound for *R* can be obtained if it is possible to specify an upper bound 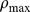 for the case detection rate and a lower bound 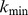 for the overdispersion parameter *k* (see Text S1). The figure shows lower and upper bound for R as a function of 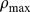. We specify 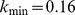 which corresponds to the SARS scenario with superspreading events.

In many situations, both the case detection rate ρ and overdispersion parameter *k* are unknown. Interestingly, 1−*F* always acts as a lower bound estimate of *R*. An upper bound for *R* can be obtained if it is possible to specify an upper bound 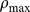 for the case detection rate and a lower bound 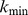 for the overdispersion parameter *k* (see Text S1). We specify 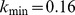 that corresponds to the SARS scenario with superspreading events. Figure 5 shows how precision decreases as the upper bound 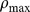 increases. However, even in the scenario 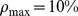, our approach can provide useful insights on transmissibility. For example, *R* is expected to be in intervals 0.20–0.25, 0.40–0.59, 0.50–0.87 for *F* = 80%, 60%, and 50%, respectively. For *F*≤46%, we can only derive a lower bound on *R*. For example, if *F* = 20%, we find that *R* is ≥0.8.

### Other Applications

Our simple estimators of transmissibility can be applied generally to study zoonoses. Nipah virus is primarily clinically characterized by fever and encephalitis and was first discovered in a large outbreak in Malaysia in 1998–1999 [17],[18]. During this outbreak (where sick pigs were believed to be the natural reservoir), Parashar et al. [19] conducted a case-control study of the risk factors for infection. They recruited patients who were hospitalized with encephalitis from January through April 1999. Candidate encephalitis patients whose serum specimen tested positive for Nipah antibody were included as cases. Other cases were detected through targeted investigations; but if we restrict analyses to the subset of patients detected through hospital surveillance, this situation resembles surveillance scenario 1 above, where the probability of being detected is independent of cluster allocation. Here, we can therefore estimate the reproduction number as *R* = 1−*G*. Probability *G* can be approximated using the data of Parashar et al. [19] by the proportion of cases who (1) either lived or worked in a farm (*G*_1_) or (2) handled a pig, or came within 1 m of a pig and came into contact with pig urine or faeces (*G*_2_). Parashar et al. [19] only give estimates for the pooled set of cases (i.e., hospital surveillance+targeted investigations) for which *G*_1_ = 95% and *G*_2_ = 92%; however, it should be straightforward to derive these statistics for the subset of cases detected through hospital surveillance alone. For *G* = 0.92–0.95, we estimate *R* = 0.05–0.08. This suggests low levels of human-to-human transmission in this first outbreak.

Subsequently, outbreaks of Nipah virus occurred in Bangladesh [20],[21]. Luby et al. identified 23 introductions of Nipah virus into human populations in central and northwestern Bangladesh from 2001 to 2007 [21]. They classified 60 of the 122 identified Nipah virus cases as reservoir-to-human infections (*G* = 49*%*). Using the simple estimator *R* = 1−*G*, we obtain *R* = 0.51. This is consistent with more detailed contact tracing data that estimated *R* = 0.48 [21]. However, both these estimators may be biased upwards as the Bengali situation corresponds to surveillance scenario 2 (i.e., detection of a case may trigger an outbreak investigation), which may lead to the selection bias discussed earlier. In such a context, we recommend running our analysis on the subset of first detected cases in each of the 23 introductions and estimating proportion *F*. Again, it should be possible to derive *F* from data collected at the time. Since the case detection rate is unknown, we expect 1−*F* to act as a lower bound for *R* (Figure 5). From the estimates of *G* and *F*, we should therefore be able to derive simple bounds on *R:* 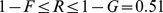.

Our approach can also be used to evaluate risks associated with non-zoonotic disease. As an example, we consider the cholera outbreak that started in the Dominican Republic in 2010 following the epidemic of that disease in Haiti. We can assess the level of local transmission in the Dominican Republic from the proportion *G* of cases detected in the Dominican Republic that were linked to Haiti (the latter country here playing the role of the natural reservoir). By December 18, 2010, three of the 59 confirmed cases detected in the Dominican Republic were linked to Haiti (*G* = 5%) [22]. Our simple estimation method cannot distinguish between *R*≈1 and *R*>1; but the observation that *G* was close to zero is indicative that transmission in the Dominican Republic was close or above levels needed for sustained human-to-human transmission.

### Combining Information from *F* and *G*

**Figure 6:**
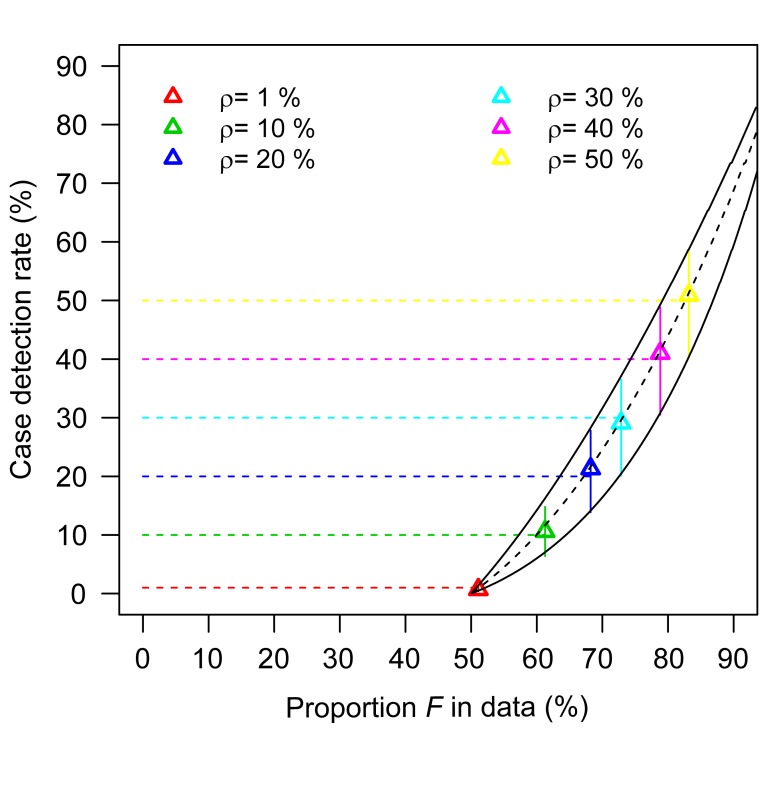
Estimating *R* and the case detection rate when both summary statistics *F* and *G* are available in surveillance scenario 1 (i.e., detection of a case does not trigger an outbreak investigation). In this simulation study, 10,000 clusters are generated for *R* = 0.5 and *k* = 0.5; six case detection rates are considered (1%, 10%, 20%, 30%, 40%, 50%; with a number of detected clusters that is 207, 1,706, 2,922, 4,092, 5,118, 6,125, respectively). First, the formula 1−*G* gives point estimates for *R* (in the range 0.48–0.51 depending on the case detection rate). For given values of *R* and the overdispersion parameter *k*, it is possible to plot the relationship between *F* and the case detection rate. Black lines in the figure correspond to *R* = 0.5 (dashed line: *k* = 0.5; plain lines: *k* = 0.16 and *k* = 5). Colour triangles show estimates of the case detection rate obtained for each dataset when *k* is assumed to be known. When *k* is unknown, vertical colour plain lines give the range of values consistent with *k* in interval 0.16–5. Horizontal colour dashed lines indicate true case detection rate.

Up to this point, in the scenario where the detection of a case did not affect detection of other cases from the same cluster (scenario 1), we assumed that cluster information was not available, and thus *F* could not be calculated. Let's now assume that such information is available and therefore both *G* and *F* can be estimated; this allows us to estimate both *R* and the case detection rate ρ, as illustrated in the simulation study shown in Figure 6. First, 1−*G* gives a point estimate for *R*. Second, once *R* is estimated, it is possible to infer the case detection rate by determining by how much proportion *F* differs from proportion *G*. If the overdispersion parameter *k* is known (e.g., from detailed contact tracing data), it is possible to accurately estimate the case detection rate; otherwise we can derive informative bounds (Figure 6).

## Discussion

We present simple methods to estimate the reproduction number of emerging zoonoses from routine surveillance data. This research project was initiated to answer a seemingly straightforward question: 50% of the H3N2v-M cases that were detected in the US in 2011 had no contact with swine. What were the implications for the level of human-to-human transmission? At the time, however, the answer did not appear as straightforward as our analysis now shows it to be, with these simple estimators *R* = 1−*G* or *R* = 1−*F*, depending on the surveillance scenario we are in.

Our approach has specific properties that potentially overcome some of the limitations of existing methods. First, the investigation effort required is less than that for other methods. For example, if there is active case finding (surveillance scenario 2), one only needs to investigate the source of infection of the first case detected in each cluster. Second, the statistical treatment of the data is extremely simple, making it possible for anyone to interpret raw surveillance statistics about the source of infection of cases (statistics *G* or *F)* in terms of human-to-human transmissibility (reproduction number *R*). Third, the method is robust to selection bias (i.e., the fact that larger clusters are more likely to be detected) and under-ascertainment (i.e., ability to detect all cases in a cluster once a cluster is identified).

However, our methods do require that the source of infection (i.e., human or natural reservoir) can be identified for either the first detected cases of a cluster or a random subset of detected cases, depending on the surveillance scenario. In the US H3N2v-M context, where most individuals have no contact with swine, it is usually relatively easy to rule out the natural reservoir as a source of infection. Determining the source of infection might however be harder in situations where a large part of the population has regular contacts with the natural reservoir (e.g., backyard poultry in rural areas). In this case, an in-depth epidemiological investigation of the potential sources of infection is required. Clearly our approach cannot be used if the zoonotic source has not been identified yet. That being said, the examples we have presented show that proxy measures for the source of transmission (e.g., contacts with the natural reservoir) are often available. Although imperfect, these proxy measures are often already used by the scientific and public health community. For example, the World Health Organization fact sheet on Nipah virus states that “in Bangladesh, half of reported cases between 2001 and 2008 were due to human-to-human transmission” [23]. Our methods improve this existing practice in at least three ways: (1) it adds an essential layer of interpretation by providing estimates of the reproduction number, *R*, which allows assessment of how far transmission is from being sustained (*R*≥1); (2) it clarifies sources of potential biases; and (3) it provides simple guidelines to reduce these biases (see summary in Box 1).

**Box 1. Approach to Assessing the Reproduction Number for Different Surveillance Scenarios**

This box summarizes the relationships between *G* (proportion of detected cases infected by the reservoir), *F* (proportion of first detected cases in each cluster infected by the reservoir), and the reproduction number *R*, for the different surveillance scenarios.

- If detection of a case does not affect detection of other cases from the same cluster (surveillance scenario 1), *R* can be estimated by *R = 1−G*. This is a general result that is independent of the case detection rate and the overdispersion parameter.
- If detection of a case may trigger an outbreak investigation (surveillance scenario 2), the selection bias that arises may lead to *R*≤1−*G*. In addition, 1−*F* always acts as a lower bound to *R*. An upper bound for *R* can be obtained if it is possible to specify an upper bound 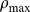 for the case detection rate and a lower bound 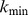 for the overdispersion parameter *k* (see Text S1). If the case detection rate is low, *R* can be estimated by 1−*F*.
- If cluster information is available in surveillance scenario 1, *R* can be estimated by *R = 1−G*. Furthermore, it is possible to estimate the case detection rate by comparing statistics *F* and *G* (see Figure 6).

If the source of infection is unknown for a subset of cases, different options are available. If data are believed to be missing at random, these cases can simply be excluded from the analysis. However, it is possible that certain sources of infection are more likely to generate missing data. In such a scenario, upper bounds for *R* can be obtained by assuming that all cases with missing data were due to human-to-human transmission and lower bounds by assuming the reverse. If human-to-human cases are prone to be classified as reservoir-to-human transmission, *R* may be underestimated. If the source of infection is uncertain, the analysis of detailed outbreak data [11],[24] might allow estimation of the probability that the case was infected by the reservoir, which could then be used to estimate *F* and *R* using our methods. Phylogenetic analysis might also help resolve uncertainties about the source of infection. Clearly, our approach will start to break down if the proportion of cases with missing data becomes too large or if the classification of cases is too unreliable. We note that other methods, for instance based on contact tracing, would also struggle in these situations.

It is possible that surveillance intensity might change over time due to increasing media attention or health concerns. In such a context, in the surveillance scenario with outbreak investigations (scenario 2), Figure 5 shows that it is possible to derive bounds on *R*. In surveillance scenario 1, a change in the case detection rate is not expected to impact the summary statistic *G* or the estimate of *R*.

A bigger source of concern is if the increase in surveillance intensity focuses disproportionately on those cases that were exposed to the natural reservoir, as this would lead to overestimating *F* and underestimating *R*. For example, with the substantial increase in H3N2v-M virus infections during the summer of 2012, CDC changed their recommendations and asked clinicians to obtain respiratory specimens from ill persons with recent swine exposure [25],[26]. Therefore, in the summer 2012, ill persons with recent swine exposure may have been more likely to have been tested for H3N2v-M infection than those without such exposure. For this reason, we cannot use our method to analyze data collected in 2012.

Nonetheless, it is interesting to note that our estimate of *R* of around 0.5 for H3N2v-M in 2011 seems larger than what is suggested by data collected in summer 2012. We believe that these differences could at least partly be explained by seasonal variations in the ability of the virus to transmit [27]. Indeed, five of the six human-to-human transmission events detected in 2011 occurred in November–December; the remaining one happened in late August.

Our method has been developed for routine surveillance systems where cases are detected independently of each other. It would require modification to be applied to data collected from cluster detection surveillance systems of the type developed after the SARS epidemic, which target unexplained clusters of severe respiratory infection [10].

While a positive property of our approach is that it does not require a full-blown outbreak investigation, there are still many good reasons to target and investigate large outbreaks. Detailed investigations of large outbreaks are indeed needed to estimate essential parameters such as transmission risk factors or the generation time with adequate power.

Our method is designed to estimate *R* in the context of subcritical outbreaks, i.e., *R*<1. As illustrated with the cholera in the Dominican Republic example, the method can give a hint that transmission is at levels close or above what is needed for sustained transmission (*R*≈1 or *R*>1). However if *R*≥1, other estimation methods are necessary to derive a point estimate of *R*.

If transmissibility of a zoonotic infection suddenly increased (for example due to seasonal factors or genetic changes in the virus), it might take time for estimates to adjust (since the method uses data from all clusters detected so far). Future developments of the method could aim to detect sudden changes in the frequency of cases linked to the reservoir, building on methods for the sequential detection of change points in quality control and dynamical systems [28]–[30].

Finally, we hope that simplicity of our method and its limited data requirements will facilitate more robust monitoring of the epidemic potential of many zoonoses known to cause occasional human case clusters (such as Crimean-Congo hemorrhagic fever, Monkeypox virus, E. coli O157∶H7, or *Mycobacterium bovis*) [12] around the world.

## Supporting Information

Figure S1 **Probability** ***F*** **that the first detected case of a cluster is infected by the reservoir as a function of the reproduction number** ***R*** **, for different assumptions on the number of chains of transmission per cluster and for different scenarios on the detection rate ρ and overdispersion in the offspring distribution** ***k*** **.** We model the number of chains per cluster with a negative binomial distribution with mean *M_L_+1* (where *M_L_ = 0*, *1*, *2*, *4*, *8*, *12*) and overdispersion *k_L_* ( = 0.1, 0.5, 1, 5), truncated to interval 0–30. The color scale gives *M_L_*.
(EPS)

Figure S2 **Log-likelihood profiles for H3N2v M, for different scenarios of detection and overdispersion in the offspring distribution.**
(EPS)

Figure S3 **Log-likelihood profiles for variant viruses other than H3N2v M, for different scenarios of detection and overdispersion in the offspring distribution.**
(EPS)

Figure S4 **Log-likelihood profiles for all strains, for different scenarios of detection and overdispersion in the offspring distribution.**
(EPS)

Figure S5 **Thinning the data.** Red line shows how estimator 1−*F* of the reproduction number changes with thinning coefficient (for example, a thinning coefficient of 30% means that 30% of cases detected in original dataset are randomly picked up for analysis). Blue line shows true value of reproduction number.
(EPS)

Table S1 **Estimates of** ***R*** **for H3N2v M, for different scenarios of detection and overdispersion in the offspring distribution.**
(DOCX)

Table S2 **Estimates of** ***R*** **for variant viruses other than H3N2v M, for different scenarios of detection and overdispersion in the offspring distribution.**
(DOCX)

Table S3 **Estimates of** ***R*** **for all strains, for different scenarios of detection and overdispersion in the offspring distribution.**
(DOCX)

Text S1 **Description of supporting information.**
(DOCX)

## Abbreviations

CDC: US Centers for Disease Control and Prevention
