# Supplementary material for: Using Routine Surveillance Data to Estimate the Epidemic Potential of Emerging Zoonoses: Application to the Emergence of US Swine Origin Influenza A H3N2v Virus
Source: PLoS Med. 2013 Mar 5;10(3):e1001399. doi: 10.1371/journal.pmed.1001399 (PMC3589342; doi:10.1371/journal.pmed.1001399)
Supplement: Table S1 — Estimates of R for H3N2v M, for different scenarios of detection and overdispersion in the offspring distribution. (DOCX) [file pmed.1001399.s006.docx]

| **Overdispersion in offspring distribution (k)** | **Case detection rate (ρ)** | **R (asymptotic)** | **R (via bootstrap)** | **Max LL** |
| --- | --- | --- | --- | --- |
| 0.16 | 1% | 0.52 [0.16,>1] | 0.54[0.17,>1] | -4.16 |
| 0.5 | 1% | 0.51[0.16,0.92] | 0.52[0.17,0.91] | -4.16 |
| 5 | 1% | 0.51[0.16,0.88] | 0.51[0.17,0.87] | -4.16 |
| 0.16 | 0.5% | 0.51[0.16,0.93] | 0.52[0.17,0.92] | -4.16 |
| 0.5 | 0.5% | 0.51[0.16,0.88] | 0.51[0.17,0.87] | -4.16 |
| 5 | 0.5% | 0.5[0.16,0.86] | 0.5[0.17,0.85] | -4.16 |
| 0.16 | 0.1% | 0.5[0.16,0.86] | 0.5[0.17,0.85] | -4.16 |
| 0.5 | 0.1% | 0.5[0.16,0.85] | 0.5[0.17,0.84] | -4.16 |
| 5 | 0.1% | 0.5[0.16,0.84] | 0.5[0.17,0.84] | -4.16 |
| 0.16 | 0.01% | 0.5[0.16,0.84] | 0.5[0.17,0.83] | -4.16 |
| 0.5 | 0.01% | 0.5[0.16,0.84] | 0.5[0.17,0.83] | -4.16 |
| 5 | 0.01% | 0.5[0.16,0.84] | 0.5[0.17,0.83] | -4.16 |
