# Supplementary material for: Using Routine Surveillance Data to Estimate the Epidemic Potential of Emerging Zoonoses: Application to the Emergence of US Swine Origin Influenza A H3N2v Virus
Source: PLoS Med. 2013 Mar 5;10(3):e1001399. doi: 10.1371/journal.pmed.1001399 (PMC3589342; doi:10.1371/journal.pmed.1001399)
Supplement: Table S3 — Estimates of R for all strains, for different scenarios of detection and overdispersion in the offspring distribution. (DOCX) [file pmed.1001399.s008.docx]

| **Overdispersion in offspring distribution (k)** | **Case detection rate (ρ)** | **R (asymptotic)** | **R (via bootstrap)** | **Max LL** |
| --- | --- | --- | --- | --- |
| 0.16 | 1% | 0.27[0.13,0.45] | 0.27[0.11,0.46] | -15.45 |
| 0.5 | 1% | 0.26[0.13,0.45] | 0.26[0.11,0.45] | -15.45 |
| 5 | 1% | 0.26[0.13,0.44] | 0.26[0.11,0.45] | -15.45 |
| 0.16 | 0.5% | 0.26[0.13,0.45] | 0.26[0.11,0.45] | -15.45 |
| 0.5 | 0.5% | 0.26[0.13,0.44] | 0.26[0.11,0.45] | -15.45 |
| 5 | 0.5% | 0.26[0.13,0.44] | 0.26[0.11,0.45] | -15.45 |
| 0.16 | 0.1% | 0.26[0.13,0.44] | 0.26[0.11,0.45] | -15.45 |
| 0.5 | 0.1% | 0.26[0.13,0.44] | 0.26[0.11,0.45] | -15.45 |
| 5 | 0.1% | 0.26[0.13,0.44] | 0.26[0.11,0.45] | -15.45 |
| 0.16 | 0.01% | 0.26[0.13,0.44] | 0.26[0.11,0.44] | -15.45 |
| 0.5 | 0.01% | 0.26[0.13,0.44] | 0.26[0.11,0.44] | -15.45 |
| 5 | 0.01% | 0.26[0.13,0.44] | 0.26[0.11,0.44] | -15.45 |
