# Supplementary material for: Using Routine Surveillance Data to Estimate the Epidemic Potential of Emerging Zoonoses: Application to the Emergence of US Swine Origin Influenza A H3N2v Virus
Source: PLoS Med. 2013 Mar 5;10(3):e1001399. doi: 10.1371/journal.pmed.1001399 (PMC3589342; doi:10.1371/journal.pmed.1001399)
Supplement: Text S1 — Description of supporting information. (DOCX) [file pmed.1001399.s009.docx]

**Supplementary Information**

**Using routine surveillance data to estimate the epidemic potential of emerging zoonoses: Application to the emergence of US swine origin influenza A H3N2v virus**

Simon Cauchemez^1^, Scott Epperson^2^, Matthew Biggerstaff^2^, David Swerdlow^2^, Lyn Finelli^2^, Neil Ferguson^1^

^1^: MRC Centre for Outbreak Analysis and Modelling, Department of Infectious Disease Epidemiology, Imperial College London, London, UK

^2^: Centers for Disease Control and Prevention, Atlanta, USA

**Contents**

[1 Small probability of detection 2](#_Toc346200384)

[2 Impact of the number of chains of transmission per cluster 2](#_Toc346200385)

[3 Comparison of asymptotic and bootstrap estimates 2](#_Toc346200386)

[3.1 H3N2v M variant 2](#_Toc346200387)

[3.1 Variant viruses other than H3N2v M 3](#_Toc346200388)

[3.2 All strains 3](#_Toc346200389)

[4 Bounds on R when the case detection rate and the overdispersion parameter are unknown (scenario 2) 3](#_Toc346200390)

[5 Thinning the data 4](#_Toc346200391)

[6 Additional discussion points 4](#_Toc346200392)

[6.1 Heterogeneity in age specific susceptibility 4](#_Toc346200393)

[6.2 Defining independent clusters 5](#_Toc346200394)

[*6.3* Confidence intervals for estimator 1-*G* (scenario 1) 5](#_Toc346200395)

[7 References 5](#_Toc346200396)

# Small probability of detection

When the probability of detection is small (*ρ<<1)*, we can approximate equation (2):

$$F\left(R,k,\rho\right)\approx\frac{\rho}{\sum_{L} \rho L.g\left(L|R,k\right)}$$

$$F\left(R,k,\rho\right)\approx\frac{1}{\sum_{L} L.g\left(L|R,k\right)}$$

$$F\left(R,k,\rho\right)\approx\frac{1}{\bar{L}}$$

where $\bar{L}$is the average length of a chain. But branching process theory tells $\bar{L}=1/\left(1-R\right)$ for a pathogen with insufficient transmissibility to cause an uncontrolled epidemic (i.e. *R*<1) [[1](#_ENREF_1)]. Hence we are left with the simple linear relationship *F*=1-*R.*

# Impact of the number of chains of transmission per cluster

As explained in the main manuscript, we define a chain of transmission as a single reservoir-to-human transmission event followed by subsequent human-to-human transmission events (if any). A cluster of related cases can be made of a few chains of transmission (i.e. when a number of people are exposed to the same zoonotic source of infection). In the manuscript, we initially assume that each cluster is made of one chain of transmission. Here, we explore sensitivity of results to this assumption.

In Figure S1, we explore how the relationship between the reproduction number *R* and the probability *F* that the first detected case of the cluster was infected by the reservoir is affected by the number of chains per cluster. We model the number of chains per cluster with a Negative Binomial distribution with mean *M_L_+1* (where *M_L_=0, 1, 2, 4, 8, 12*) and overdispersion *k_L_* (=0.1, 0.5, 1, 5), truncated to interval 0-30 [NB: *M_L_*=0 corresponds to a cluster made of 1 chain of transmission]. We find that our results are largely robust to the presence of multiple chains per cluster (Figure S1). For example, if *ρ*=1%, *R*=1.0, *k*=5, as *M_L_* changes from 1 to 4, *F* changes from 8% to 11%.

# Comparison of asymptotic and bootstrap estimates

## H3N2v M variant

Three out of 6 first detected cases were infected by the reservoir in H3N2v M clusters. Estimates of *R* for H3N2v M are presented in Table S1 for different scenarios of detection and overdispersion in the offspring distribution.

## Variant viruses other than H3N2v M

Seventeen out of 21 first detected cases were infected by the reservoir in clusters of strains that were not H3N2v M. Estimates of *R* for variant viruses other than H3N2v M are presented in Table S2 for different scenarios of detection and overdispersion in the offspring distribution.

## All strains

Twenty out of 27 first detected cases were infected by the reservoir in all the detected clusters. Estimates of *R* for all strains are presented in Table S3 for different scenarios of detection and overdispersion in the offspring distribution.

# Bounds on R when the case detection rate and the overdispersion parameter are unknown (scenario 2)

Consider the statistical framework for surveillance scenario 2. Assume that the case detection rate $\rho$and the overdispersion parameters *k* are unknown but that we can put boundaries on these parameters: $\rho_{\min}\leq\rho\leq\rho_{\max}$and$k_{\min}\leq k\leq k_{\max}$. The estimator of the reproduction number $\tilde{R}_{f,\rho,k}$is a function of $\rho$ and *k* as well as the observed proportion *f* of first detected cases infected by the reservoir. An interesting property of our estimator that we demonstrate below is that:

$$\tilde{R}_{\left(f,\rho_{\min},k_{\max}\right)}\leq\tilde{R}_{\left(f,\rho,k\right)}\leq\tilde{R}_{\left(f,\rho_{\max},k_{\min}\right)}$$

For lower bound$\rho_{\min}$ small enough, we can simplify to

$$1-f\leq\tilde{R}_{\left(f,\rho,k\right)}\leq\tilde{R}_{\left(f,\rho_{\max},k_{\min}\right)}$$

If we are able to define an upper bound on the case detection rate and a lower bound on the overdispersion parameter, then we derive an interval $\left[1-f,\tilde{R}_{\left(f,\rho_{\max},k_{\min}\right)}\right]$ of values for *R* that are consistent with observed proportion *f*. In the applications, we use the SARS overdispersion parameter *k_min_*=0.16 as the lower bound for *k*.

We are now going to demonstrate that$\tilde{R}_{\left(f,\rho,k\right)}\leq\tilde{R}_{\left(f,\rho_{\max},k_{\min}\right)}$.

We first note the 3 following inequalities

- If $R_{1}\leq R_{2}$, $F\left(R_{2},\rho,k\right)\leq F\left(R_{1},\rho,k\right)$ (S1)
- If $\rho_{1}\leq\rho_{2}$,$F\left(R,\rho_{1},k\right)\leq F\left(R,\rho_{2},k\right)$ (S2)
- If $k_{1}\leq k_{2}$, $F\left(R,\rho,k_{2}\right)\leq F\left(R,\rho,k_{1}\right)$ (S3)

Inequality (S2) can be demonstrated based on the derivation of *F* with respect to *ρ*:

$$\frac{\partial F}{\partial\rho}=\frac{\sum_{L} \left(1-\left(1-\rho\right)^{L-1}\right).g\left(L|R,k\right)}{\left(\sum_{L} \left(1-\left(1-\rho\right)^{L}\right).g\left(L|R,k\right)\right)^{2}}>0$$

Given the complex expression of the distribution of cluster size (equation 1), we perform an extensive exploration of the parameter space to validate inequalities (S1) and (S3) with *R*=0.01,0.02,…,0.95; *ρ*=0.01,0.02,…,0.80; *k*=0.10,0.11,…,5.

Then, given *f* the observed proportion of first detected cases infected by the reservoir, parameters *ρ* and *k*, the point estimate$\tilde{R}_{\left(f,\rho,k\right)}$ for the reproduction number is such that$f=F\left(\tilde{R}_{\left(f,\rho,k\right)},\rho,k\right)$

With inequalities (S2) and (S3), we obtain

$f=F\left(\tilde{R}_{\left(f,\rho,k\right)},\rho,k\right)\leq F\left(\tilde{R}_{\left(f,\rho,k\right)},\rho_{\max},k_{\min}\right)$ (S4)

But since *F* is a decreasing function of *R* (inequality S1),$\tilde{R}_{\left(f,\rho_{\max},k_{\min}\right)}$must satisfy $\tilde{R}_{\left(f,\rho,k\right)}\leq\tilde{R}_{\left(f,\rho_{\max},k_{\min}\right)}$to satisfy both inequality (S4) and definition$f=F\left(\tilde{R}_{\left(f,\rho_{\max},k_{\min}\right)},\rho_{\max},k_{\min}\right)$.

# Thinning the data

When the estimator 1-*F* is used, we show in the main manuscript that surveillance intensity (i.e. the case detection rate) impacts bias, and that lower detection rates result in lower bias (Figure 4a). There may therefore be a value in downsampling observations. We have performed a simulation study to test this possibility. We consider the surveillance scenario *“Routine sentinel surveillance alone”* where it is assumed that a case can be linked to a cluster. We generated 100,000 clusters with *R*=0.5 and *k*=0.5; and considered 4 case detection rates (20%, 30%, 40%, 50%). Figure S5 shows the estimator *R*=1-*F* for different levels of thinning of the data (from 1% to 90%). If thinning is sufficiently important, estimator *R*=1-*F* becomes unbiased.

However, it should be noted that in this particular surveillance scenario, a simpler approach can be used. This is the approach presented in result section “Combining information from *F* and G” and Figure 6.

# Additional discussion points

## Heterogeneity in age specific susceptibility

Our method does not allow estimating all the parameters of an age-structured epidemic model, in particular age-specific relative susceptibilities. Nonetheless, even in a context of marked heterogeneity of susceptibility by age (as apparent for H3N2v M virus), the method should provide an unbiased estimate of the reproduction number, defined as the dominant eigenvalue of the next generation matrix. We note that the same applies to other estimation methods that rely on case-only data, for example the epidemic curve [[2](#_ENREF_2)] or contact tracing data [[3](#_ENREF_3)]. In order to be able to assess age-specific susceptibilities, one needs data on cases, but also on members of the population that were not infected [[4](#_ENREF_4),[5](#_ENREF_5)].

## Defining independent clusters

When detection of a case does not affect the detection probability of other cases of the same cluster (scenario 1), *R* can be estimated by 1-*G*. This does not require information on clusters. In contrast, in the scenario with outbreak investigations (scenario 2), cluster allocation is needed to control for selection bias; and the different clusters need to be independent of each other. Consider the following example. After an outbreak investigation, there is evidence that the cluster was made of few chains of transmission (e.g. a number of people are infected by the zoonotic source in a fair) and that each case can be allocated to one of the chains of transmission. Here, the different chains of transmission are not independent of each other (because they were detected during the same investigation); so they should be seen as a single cluster. In section 1 of the Supplementary Information, we show that the relationship between *R* and *F* is relatively robust to assumptions about the number of chains of transmission per cluster.

## Confidence intervals for estimator 1-*G* (scenario 1)

The precision of estimator 1-*G* may be affected by variations at the population level and at the level of the sample of detected cases.

First, at the population level, let $N_{I}$ denote the total number of chains of transmission (both detected and undetected) in the study population and $N_{T}$ denote the total number of cases in that population. The expectation of the ratio $N_{I}/N_{T}$is 1-*R* (see Methods).

Second, consider the sample of *M* cases that are detected and assume that *m* of them were infected by the reservoir. We have:

$m\sim Bin\left(M,\frac{N_{I}}{N_{T}}\right)$ (S5)

which we approximate by the likelihood:

$m\sim Bin\left(M,1-R\right)$ (S6)

This is valid if the total number of chains in the population is large enough so that we can assume$N_{I}/N_{T}$is equal to its expected value 1-*R*. If this not the case, confidence bounds may underestimate uncertainty.

# References

1. Ferguson NM, Fraser C, Donnelly CA, Ghani AC, Anderson RM (2004) Public health. Public health risk from the avian H5N1 influenza epidemic. Science 304: 968-969.

2. Wallinga J, Teunis P (2004) Different epidemic curves for severe acute respiratory syndrome reveal similar impacts of control measures. Am J Epidemiol 160: 509-516.

3. Lloyd-Smith JO, Schreiber SJ, Kopp PE, Getz WM (2005) Superspreading and the effect of individual variation on disease emergence. Nature 438: 355-359.

4. Cauchemez S, Bhattarai A, Marchbanks TL, Fagan RP, Ostroff S, et al. (2011) Role of social networks in shaping disease transmission during a community outbreak of 2009 H1N1 pandemic influenza. Proceedings of the National Academy of Sciences of the United States of America 108: 2825-2830.

5. Cauchemez S, Donnelly CA, Reed C, Ghani AC, Fraser C, et al. (2009) Household transmission of 2009 pandemic influenza A (H1N1) virus in the United States. New England Journal of Medicine 361: 2619-2627.
